# Inflammatory Choroidal Neovascular Membranes: Clinical Profile, Treatment Effectiveness, and Visual Prognosis

**DOI:** 10.1155/2021/9982883

**Published:** 2021-07-23

**Authors:** Gonçalo Carrola, Mário Lima-Fontes, Fernando Falcão-Reis, Luís Figueira, Ângela Carneiro

**Affiliations:** ^1^Faculty of Medicine, University of Porto, Porto, Portugal; ^2^Department of Ophthalmology, São João Hospital University Centre, Porto, Portugal; ^3^Departments of Surgery and Physiology, Faculty of Medicine, University of Porto, Porto, Portugal; ^4^Department of Pharmacology and Therapeutics, Faculty of Medicine of the University of Porto, Porto, Portugal; ^5^Centre for Drug Discovery and Innovative Medicines (MedInUP), University of Porto, Porto, Portugal

## Abstract

**Purpose:**

To characterise a sample of patients with inflammatory choroidal neovascularization (I-CNV), including clinical profile, underlying aetiology and its course, treatments performed, associated clinical response, and visual prognosis.

**Methods:**

Retrospective analysis of patients with a diagnosis of I-CNV followed at the Ophthalmology Department of *Centro Hospitalar Universitário de São João* (CHUSJ). Clinical and visual outcomes were classified according to the difference in visual acuity after treatment.

**Results:**

Twenty eyes from 17 patients were analysed (11 female and 6 male patients, mean age 41.90 ± 16.457 years at CNV diagnosis). Punctate inner choroidopathy/multifocal choroiditis was the predominant inflammatory aetiology (10 patients, 58.82%). Median follow-up time was 46 months (range 10 to 188 months). Neovascularization was treated with intravitreal anti-VEGF injections (bevacizumab, aflibercept, and ranibizumab), and inflammation with anti-inflammatory/immunosuppressive therapy (oral, intravenous, and/or intravitreal corticosteroids; oral cyclosporine or methotrexate). Intravitreal anti-VEGF agents had a median number of 7.00 injections (IQR, 4.25 to 29.00). Visual acuity among 20 eyes had a mean gain of 15.10 ± 12.998 ETDRS letters after anti-VEGF treatment (*p*=0.000051). According to our classification, 16 had an improved outcome (80.00%), 3 had a stable outcome (15.00%), and 1 had a worsened visual outcome (5.00%). In addition, 13 eyes (65.00%) had a final VA equal to or greater than 65 letters. Recurrence was seen in 3 eyes (15.00%). Complications included cataract (6 patients) and ocular hypertension (4 patients).

**Conclusion:**

A combined approach with anti-VEGF agents and anti-inflammatory therapy was effective in I-CNV treatment, and an overall good visual prognosis was attainable. Intensive follow-up was fundamental in the management of both the primary inflammatory and secondary neovascular conditions.

## 1. Introduction

Inflammatory choroidal neovascularization (I-CNV) is characterised by the pathologic growth of blood vessels from the choroid to the subretinal and/or subretinal pigment epithelium spaces due to an inflammatory cause [1, 2]. In fact, inflammation represents the third most common cause of choroidal neovascularization (CNV), behind age-related macular degeneration (AMD) and pathologic myopia [1]. Regardless of the aetiology, what these entities have in common is a disruption in Bruch's membrane combined with an inflammatory and angiogenic cascade [3]. Endothelial and inflammatory cell invasion leads to the formation of neovascular membranes with associated leakage and haemorrhage and, consequently, a variable degree of vision loss [1, 3].

Inflammatory causes of CNV can be mainly divided into infectious and noninfectious uveitis (Table 1) [1].

Nevertheless, the risk of developing CNV in patients with these conditions varies considerably [4]. I-CNV is better documented in noninfectious than in infectious etiologies [1]. In addition, specific morphologic syndromes appear to be more correlated to this complication in comparison with eyes with nonsyndromic panuveitis or posterior uveitis [4]. Punctate inner choroidopathy, multifocal choroiditis, and Vogt–Koyanagi–Harada disease are some of those syndromes with higher incidences of CNV [4].

Even though CNV is a relatively rare complication of inflammatory diseases, it can result in severe vision loss and it has a big impact on patients' work ability and productivity, since it happens commonly during active years [4–6]. This correlates with the incidence of uveitis, which also tends to affect a younger population [4]. Usual presenting symptoms are distortion and metamorphopsia, which can be accompanied by decreased visual acuity or scotoma [1]. Yet, many patients are asymptomatic, so new-onset neovascular membranes are often diagnosed by imaging techniques during follow-up of their inflammatory condition [4].

Diagnosis still remains a challenge, but prompt treatment initiation is essential in order to avoid possibly rapid and irreversible visual loss [1, 6, 7]. Traditional fluorescein angiography and indocyanine green angiography coupled with optical coherence tomography have been crucial to not only ensure an accurate and early diagnosis but also to monitor disease progression and treatment response. Optical coherence tomography angiography is a recently used complementary imaging modality, also useful in the diagnosis and management of this condition [1, 7].

Management of inflammatory choroidal neovascularization has had massive progress in recent years/decades. Introduction of antivascular endothelial growth factor (VEGF) intravitreal therapies seems to be a major determinant decreasing neovascularization and, consequently, improving visual acuity [8, 9]. Such evidence agrees with the practically consensual understanding of VEGF as a key mediator in CNV pathogenesis [3]. In fact, in I-CNV, there are two disease processes we aim to control: neovascularization itself and the underlying inflammatory cause. It is consensual nowadays that a combined approach with both local and systemic therapies results in better disease control and overall visual prognosis. Accordingly, intravitreal anti-VEGF agents, such as bevacizumab, aflibercept, and ranibizumab, in association with systemic immunosuppressive/anti-inflammatory drugs, with or without corticosteroids, are now the standard of care for these patients. As a matter of fact, directly addressing the inflammatory environment, which is itself the root of I-CNV formation, will probably terminate the angiogenic drive, therefore leading to better outcomes [1, 5]. Other therapies, such as photodynamic therapy (PDT) with verteporfin, can also be used to manage the disease [1].

Anti-VEGF therapy seems promising treating I-CNV, as reported by several small sample size studies. However, there is a paucity of clinical trials regarding this theme and no well-accepted anti-VEGF treatment algorithm for I-CNV [5].

In this sense, the purpose of this study is to characterise a sample of patients followed at *Centro Hospitalar Universitário de São João* (CHUSJ) with inflammatory choroidal neovascularization including clinical profile, underlying aetiology of I-CNV and its course, treatments performed and associated clinical response, and visual prognosis.

## 2. Methods

### 2.1. Study Design

An observational retrospective case series was conducted in a tertiary care centre group of patients.

### 2.2. Study Population

**Figure 1:**
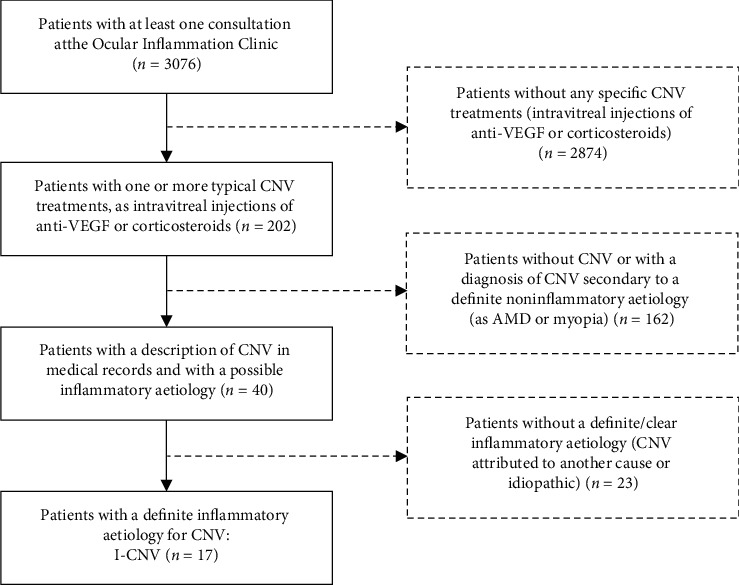
Process of patient's selection.

Patients with a diagnosis of inflammatory choroidal neovascularization followed at the Ophthalmology Department of CHUSJ were identified as described in Figure 1.

A list of patients followed at the Ocular Inflammation Clinic since January 2008 until November 2020 was generated. From the 3076 patients obtained, 202 were submitted to intravitreal treatments and were selected for medical records analysis.

Criteria for inclusion were as follows:

- Diagnosis of CNV established by fundoscopy, optical coherence tomography, fluorescein angiography, and indocyanine green angiography
- Diagnosis of an underlying inflammatory eye/systemic condition

Patients without CNV or with CNV secondary to other causes were excluded.

Thus, our final population included 17 patients. They were numerically ordered according to CNV diagnosis, from oldest to most recent (P1 to P17).

Data were collected retrospectively from medical records, including age, sex, past medical history, regular medication, refractive errors, underlying inflammatory disease characterisation (aetiology, symptoms/signs, and date of diagnosis), CNV characterisation (affected eyes, symptoms/signs, and date of diagnosis), complications, recurrence, other eye diseases/problems, imaging exam descriptions, treatments (directed to the inflammatory cause and CNV), and visual acuity.

Currently, no agreement exists in respect to punctate inner choroidopathy (PIC) and multifocal choroiditis (MFC) classification as separate entities or belonging to the same spectrum of disease [10–12]. Therefore, and because they are difficult to differentiate, we classified these entities as a common aetiology group in our study: PIC/MFC.

### 2.3. Treatment

Treatment of neovascularization in our care centre included photodynamic therapy with verteporfin until 2006. Afterwards, CNV was addressed with intravitreal anti-VEGF agents: bevacizumab, aflibercept, and ranibizumab.

Inflammation was treated with anti-inflammatory/immunosuppressive agents according to disease severity and patient's response to treatment. Anti-inflammatory therapy included oral, intravenous, and/or intravitreal corticosteroids, and immunosuppressants used were cyclosporine or methotrexate.

### 2.4. Outcome Definition

Best-corrected visual acuity (BCVA) was determined using the Early Treatment Diabetic Retinopathy Study (ETDRS) charts. Four visual acuities were defined for each patient:

1. Baseline VA: at primary inflammatory/infectious disease presentation or beginning of follow-up
2. Pretreatment VA (pre-Tx VA): at CNV presentation, prior to the beginning of intravitreal anti-VEGF therapy
3. Posttreatment VA (post-Tx VA): after intravitreal anti-VEGF therapy suspension and VA stabilisation
4. Final VA: last available VA during follow-up (until November 2020)

Whenever both primary inflammatory and CNV presentations/diagnosis were simultaneous, baseline VA was equal to pretreatment VA. When patients maintained intravitreal anti-VEGF treatment at the last evaluation, final VA was the same as posttreatment VA.

Visual outcome was characterised according to the difference in visual acuity after intravitreal anti-VEGF treatment. For this purpose, a new variable called deltaVA (ΔVA) representing the difference between posttreatment VA and pretreatment VA was created (difference in absolute number of letters).

An outcome higher than or equal to 5 letters was considered improved, an outcome between −4 and 4 letters was counted as stable, and an outcome equal to or lower than −5 letters was classified as worsened.

### 2.5. Statistical Analysis

Descriptive statistics were performed to describe clinical data (mean, standard deviation (SD), median, interquartile range (IQR), and frequency).

Several nonparametric Mann–Whitney *U* tests were performed when comparing categorical variables (cataract, PIC/MFC, infectious or noninfectious inflammatory aetiology, CNV recurrence, and anti-VEGF treatment suspension) and nonnormally distributed quantitative variables (total number of anti-VEGF injections). The mentioned categorical variables were also compared with normally distributed quantitative variables (age, pretreatment VA, posttreatment VA, and deltaVA) using several independent *T*-tests.

Quantitative variables (spherical equivalent, pretreatment VA, posttreatment VA, and deltaVA) were analysed using Pearson's correlation coefficient. Categorical variables were analysed using chi-square tests.

Statistical analysis was performed using IBM SPSS Statistics for Windows, version 27.0 (IBM Corp., Armonk, NY), and statistical significance was defined with a *p* value inferior to 0.05.

### 2.6. Ethics Statement

This study, including all the applied methodology, was approved by the Ethics Committee of CHUSJ/FMUP in October 2020.

## 3. Results

### 3.1. Sample Description

In this study, 17 patients were included. The majority, 14 patients, had only one eye affected with CNV (82.35%), and 3 had both eyes affected (P5, P11, and P12; 17.65%). Thus, a total of 20 affected eyes were analysed. From those with unilateral involvement, 8 were on the right side and 6 on the left side.

Our study population was predominantly female, accounting for 11 female patients (64.71%) and 6 male patients (35.29%). The mean age of patients at CNV diagnosis among the 20 affected eyes was 41.90 ± 16.457 years, ranging between 20 and 69 years. Specific epidemiological details are summed up in Table 2.

Additionally, 7 patients (P2, P3, P4, P5, P8, P11, and P12) had concomitant high myopia, defined as spherical equivalent ≤ -6.00 dioptres. Because P5, P11, and P12 had bilateral I-CNV, a total of 10 eyes were highly myopic.

Baseline spherical equivalent was not possible to obtain for patients P6 and P9, due to previous refractive surgery in other healthcare facilities (phacoemulsification with intraocular lens implantation and LASIK surgery, respectively). Median spheric equivalent of the remaining selected eyes was −9.50 dioptres.

Other eye pathology, general history, and baseline eye refraction history are seen in Supplementary Table 1 (see Supplementary Material).

### 3.2. CNV Aetiology

CNV was secondary to a noninfectious aetiology in 15 patients (88.24%) and to an infectious cause in 2 patients (11.76%).

The noninfectious group comprehended 10 patients with the entity punctate inner choroidopathy/multifocal choroiditis (58.82%), 2 patients with sarcoidosis (11.76%), 1 patient with an ambiguous diagnosis between multifocal choroiditis and serpiginous choroiditis (5.88%), 1 patient with serpiginous choroiditis (5.88%), and 1 patient with Vogt–Koyanagi–Harada disease (5.88%).

The infectious aetiology subgroup comprehended 1 patient with toxoplasmosis (5.88%) and 1 patient with nocardiosis (5.88%).

Aetiology information is summed up in Table 2.

From 15 noninfectious patients, most were female: 11 females *versus* 4 males. In contrast, the 2 infectious cases were both male.

Since punctate inner choroidopathy/multifocal choroiditis was by far the predominant I-CNV cause (10 out of 17 patients), patients with this aetiology were compared with the remainder. The mean age at CNV presentation was inferior in PIC/MFC patients than in other patients (39.46 ± 15.554 *versus* 46.43 ± 18.356; *p*=0.381). The proportion of female patients with PIC/MFC was also higher when compared to the remaining patients (70.00% vs. 57.14%, *p*=0.644).

### 3.3. Presentation and Follow-Up

Most patients presented with typical CNV symptoms, such as metamorphopsia, vision loss, and scotoma (17 out of 20 eyes, 85.00%). The other 3 eyes were only diagnosed with CNV due to suggestive imaging findings, despite patients being asymptomatic (eyes E5.2, E6, and E17, 15.00%).

For instance, P5 was previously diagnosed with symptomatic CNV secondary to PIC/MFC on the right eye in 2010 (E5.1). During follow-up, in 2017, OCT examination showed evidence of new-onset neovascular membrane in the contralateral eye (E5.2; left), despite having no symptoms. P6 had a history of bilateral posterior uveitis secondary to sarcoidosis and was being followed at the Ocular Inflammation Clinic. His visual acuity before CNV diagnosis was low (35 letters). New symptoms were not reported by the patient, and CNV was only found over examination using OCT. Finally, P17 was diagnosed with retinochoroiditis due to Nocardia infection (*Nocardia abscessus*). During follow-up, OCT showed new-onset CNV membrane on the left eye without reported symptoms.

All patients except P14 had a posterior uveitis at presentation. P14 presented with an intermediate uveitis, but later developed posterior manifestations.

Neovascular membranes were also classified according to location as subfoveal, juxtafoveal, or peripapillary (Table 2). Lesions were mostly subfoveal, affecting 12 eyes (60.00%), followed by juxtafoveal location, with 7 eyes (35 00%), and only 1 eye with peripapillary CNV location (5.00%).

Median follow-up time since CNV diagnosis was approximately 46 months (45.50 (IQR, 40.00 to 111.25)), ranging between 10 and 188 months. Two patients had a previous I-CNV diagnosis and follow-up in another care centre (P9 and P13). They then continued treatment and follow-up in our medical centre. Only one patient lost follow-up after treatment (P10). Nevertheless, posttreatment visual acuity was available on medical records and was stabilised before the patient discontinued the consultations, so the anti-VEGF effect in his eye was analysed.

### 3.4. Local Therapy: PDT and Anti-VEGF

Two patients were previously treated with photodynamic therapy (PDT), specifically P1 and P2, both in 2005. PDT was used in our care centre before anti-VEGF treatment, and these two patients were the first diagnosed with I-CNV among our sample.

All patients were treated with intravitreal anti-VEGF injections at some point. Anti-VEGF agents used in CHUSJ included bevacizumab, aflibercept, and ranibizumab. The total number of intravitreal injections differed considerably, from 2 to 111, and had a median of 7.00 (IQR, 4.25 to 29.00) among the twenty included eyes. Most eyes, 12 specifically, were only treated with bevacizumab, 5 eyes were treated with two different anti-VEGF agents (bevacizumab and aflibercept in 4 eyes; bevacizumab and ranibizumab in 1 eye), and 3 eyes were treated with all three agents (bevacizumab, aflibercept, and ranibizumab). Therefore, bevacizumab was used in all 20 eyes, aflibercept in 7 eyes, and ranibizumab in 4 eyes. Specific anti-VEGF and number of injections for each eye are shown in Table 3.

At the end of the study, 6 patients maintained intravitreal treatment with anti-VEGF (35.29%). P5 maintained treatment in both eyes, so 7 eyes were still on anti-VEGF treatment (Table 3).

A significant statistical association was found between the development of cataract and the median number of anti-VEGF intravitreal injections (35.00 injections in patients with cataract (IQR, 6.00 to 42.00) *versus* 7.00 injections in patients without cataract (IQR, 3.00 to 8.50), *p*=0.031).

The median total of anti-VEGF injections was equal between the noninfectious and the infectious aetiology groups, both having a median of 7.00 injections. Median total anti-VEGF injections between eyes with and without PIC/MFC also did not differ, respectively (7.00 (IQR, 3.50 to 36.00) *versus* 7.00 (IQR, 6.00 to 10.00), *p*=0.661).

### 3.5. Systemic Treatment

From all 17 patients, 10 received oral corticosteroids (58.82%).

Only one patient had intravenous corticosteroids (P16, 5.88%). He had oral and intravenous prednisolone in Brazil to treat Vogt–Koyanagi–Harada disease. One patient (P8, 5.88%) was treated with intravitreal CCTs (triamcinolone) to help control PIC/MFC activity.

Oral immunosuppressive therapy was used in 6 patients (35.29%): 5 (P4, P8, P11, P12, and P13) had cyclosporin and 1 (P14) had methotrexate. P4 changed immunosuppressive treatment from cyclosporin to azathioprine because of cyclosporine adverse effects. He afterwards initiated treatment with biologic therapy using adalimumab due to severe refractory disease and high risk of central vision loss. He was the only patient in our study receiving biologic therapy.

Absence of systemic treatment can be explained, on the one hand, because the inflammatory condition stayed inactive during follow-up in some patients. On the other hand, in patients belonging to the infectious group, direct treatment of infection would almost certainly control the inflammatory process. In fact, none of the two patients (P15 and P17) belonging to the infectious aetiology subgroup were treated with corticosteroids.

Regarding the infectious etiologies, P15 did not receive specific therapy to treat toxoplasmosis, since this condition was inactive at presentation and during follow-up. In contrast, P17 presented with a recrudescence from a previous Nocardia infection, this time with eye involvement. An anti-Nocardia therapy was used, specifically a combined therapy of trimethoprim-sulfamethoxazole (TMP-SMX) and a beta-lactam antibiotic. The patient was hospitalised for systemic study, so he underwent intravenous treatment with TMP-SMX and a carbapenem firstly and ceftriaxone secondly. Oral treatment was then continued for 12 months with TMP-SMX and cefixime.

All therapeutic measures are summed up in Table 3.

### 3.6. Visual Outcome

The mean deltaVA among the 20 eyes was +15.10 ± 12.998 (*p*=0.000051 (range: −8 to +49)). In other words, patients had a mean gain of approximately 15 letters in their visual acuity. Mean pretreatment visual acuity was 50.85 ± 19.148 letters, and mean posttreatment visual acuity was 65.95 ± 19.168. Posttreatment VA had a significant positive strong correlation to the pretreatment VA (Pearson correlation coefficient = 0.770, *p*=0.000072). These results are shown in Table 4.

According to our classification, 16 eyes had an improved outcome (80,00%), 3 had a stable outcome (15.00%), and 1 had a worsened visual outcome (E13, 5.00%). In addition, 13 eyes (65.00%) had a final VA equal to or greater than 65 letters.

The mean baseline VA was 62.15 ± 22.203 letters, and the mean final VA was 63.45 ± 21.508 letters. Individual visual acuities and deltaVA are described in Table 5.

When analysing visual outcomes in accordance with the aetiology group, the mean deltaVA was +15.00 ± 13.668 letters in the noninfectious subgroup and +16.00 ± 5.657 letters in the infectious subgroup (*p*=0.921). No associations were also found for pre-Tx VA (*p*=0.961) and post-Tx VA (*p*=0.908) between the abovementioned subgroups.

No significant statistical differences were found when comparing pre-Tx VA (*p*=0.813), post-Tx VA (*p*=0.975), and deltaVA (*p*=0.763) between eyes of patients with PIC/MFC and those with other etiologies.

### 3.7. Recurrence and Complications

Recurrence of I-CNV pathology was seen in 3 eyes (E2, E5.1, and E15; 15.00%). Management of these cases was made using further anti-VEGF treatment with bevacizumab. Other 5 eyes, although not classified as recurrent, maintained active I-CNV during all follow-up period, not being able to suspend anti-VEGF treatment (E1, E4, E5.2, E13, and E17; 25.00%). DeltaVA was not significantly related to CNV recurrence (*p*=0.436) or treatment suspension (*p*=0.408).

Patient 17 had only 10 months of follow-up, so more time would be needed to evaluate the possibility to discontinue treatment if stabilisation was achieved. Follow-up time was longer than 3 years in the other four cases (E1, E4, E5.2, and E13 with 188, 126, 46, and 45 months, respectively). However, neovascular membranes were still active, and anti-VEGF treatment suspension was not possible.

Complications included cataract and ocular hypertension. Cataract was seen in 6 patients (P1, P3, P5, P6, P13, and P14). Five had bilateral cataract and one (P13) had unilateral on the left side. Three patients underwent phacoemulsification with intraocular lens implantation (P5, P6, and P14).

Four patients (P4, P6, P11, and P14) developed ocular hypertension. All were treated using topical medication. P16 also underwent trabeculectomy and cyclophotocoagulation.

Details about CNV recurrence, treatment suspension, and complications are described in Supplementary Tables 2 and 3 (see Supplementary Material).

## 4. Discussion

Inflammatory choroidal neovascularization is an uncommon yet sight-threatening complication of ocular inflammatory diseases. It represents a diagnostic and treatment challenge, due to the heterogeneity in associated etiologies, array of presentations and disease courses, and difficulty in differentiating between inflammatory choroidal lesions and choroidal neovascularization. In contrast with neovascular AMD, there are no long-term studies reporting treatment efficacy and visual prognosis in I-CNV. Therefore, research studies aiming to describe and analyse patients with this condition are fundamental to understand the best clinical approach and management.

Here we described a group of 17 patients with a total of 20 eyes affected with I-CNV and compared their clinical profiles, treatments performed, and visual outcomes. Our sample was young at I-CNV presentation (mean age of approximately 40 years), predominantly female (approximately 65%), and highly myopic (median refractive error of −9.50 D). In accordance with these features, punctate inner choroidopathy/multifocal choroiditis was the most common inflammatory aetiology for CNV. Other studies have reported a higher incidence of PIC/MFC in a young myopic female population [10, 13]. Other noninfectious CNV causes found in our study were sarcoidosis, serpiginous choroiditis, and Vogt–Koyanagi–Harada disease.

In contrast, we described two cases of infectious CNV, one secondary to toxoplasmosis and the other to nocardiosis, both being male. Some studies have suggested the existence of geographical variation in inflammatory conditions leading to I-CNV, especially regarding infectious causes [1, 14]. Therefore, specific studies are important to understand the local range of I-CNV etiologies.

Practically all patients, except for one, presented with posterior uveitis. Other studies have reported CNV occurring more commonly in posterior and panuveitis [4]. Choroidal neovascularization had a predominant subfoveal location (12 eyes), followed by juxtafoveal (7 eyes) and peripapillary locations (1 eye).

I-CNV had a symptomatic presentation in the majority of the affected eyes (85%). Reported symptoms in our patients were compatible with typical CNV symptoms, for instance, metamorphopsia, vision loss, and scotoma [1]. This high incidence of CNV-related symptoms may be associated with the predominant subfoveal location of the neovascular membranes, in addition to the low mean age of the sample and alleged good previous visual acuities. Nevertheless, some eyes with I-CNV were only found through ancillary imaging, while they were being followed either for their primary inflammatory condition or for CNV in the contralateral eye. As a matter of fact, an active inflammatory state and a prior CNV diagnosis in the contralateral eye have been reported as increasing risk factors for CNV development [4]. Therefore, patients with ocular inflammatory conditions or already with I-CNV in the contralateral eye must have a tight follow-up to guarantee prompt diagnosis and treatment.

Adequate individualised treatment prevents disease progression and additional visual loss [5]. In fact, if choroidal neovascularization is adequately and opportunistically addressed and inflammatory pathology is concomitantly managed, it is possible to stabilise or even improve patient's visual acuity from their nadir at the time of CNV presentation/decompensation [5, 8]. In our study, CNV was approached with intravitreal anti-VEGF injections and uveitis was treated with anti-inflammatory/immunosuppressive agents (i.e., corticosteroids and cyclosporin). Several studies have shown the benefit of intravitreal anti-VEGF therapy in patients with CNV secondary to various inflammatory aetiologies. For instance, a retrospective multicentre case series of patients with I-CNV was one of the early and largest studies demonstrating the benefit of bevacizumab in CNV regression and significant visual improvement. In this study, from 76 eyes with I-CNV, 58 improved, 15 registered no change, and 3 worsened their visual acuities after intravitreal bevacizumab. Additionally, visual improvement was significant in punctate inner choroidopathy, multifocal choroiditis, and Vogt–Koyanagi–Harada disease [15].

There are few clinical trials regarding I-CNV and no well-established anti-VEGF treatment algorithm. The phase 3 Minerva study, a 12-month double-masked randomized clinical study for ranibizumab efficacy and safety in patients with CNV secondary to uncommon causes, including inflammation, showed clinically significant visual improvement of ranibizumab against placebo at month 2. Even though the study comprehended a wide variety of etiologies, with few patients with an inflammatory cause, it suggested that ranibizumab is effective and safe in CNV secondary to causes other than AMD and myopia, regardless of the primary aetiology [16].

We found a wide variation in the number of performed intravitreal anti-VEGF injections (range: 2 to 111, median: 7 injections). Some patients were able to stabilise the disease with a smaller number of injections, whereas others needed a substantially greater number. I-CNV recurrence was seen in 15% of eyes. This percentage seems to be lower than in myopic CNV or neovascular AMD [17–20]. In fact, since CNV was secondary to an inflammatory aetiology, managing the primary disease with an anti-inflammatory/immunosuppressive approach allows better disease control, reducing recurrence and need for anti-VEGF injections. In comparison, in a previous retrospective study of CNV secondary to PIC, the authors compared 14 eyes treated with ranibizumab monotherapy with 10 eyes treated with a combination of corticosteroids and ranibizumab. Patients treated with a combined approach received fewer ranibizumab injections, had a greater mean visual acuity improvement, and never recurred, compared with the ranibizumab monotherapy group [21].

Additionally, 25% of eyes maintained treatment during all follow-up period, never suspending anti-VEGF therapy. In one of these cases, I-CNV had been diagnosed only 10 months before, so we can speculate that more time would be needed to achieve CNV remission. All the other 4 cases had more than 3 years of follow-up. This significant variability between patient's responses to treatment emphasises the importance of an individual on demand approach, which had also been proven effective in other studies [8, 9, 22].

Follow-up in our care centre required frequent consultation with continuous patient evaluation. This allowed detection and immediate management of active CNV through patients *de novo* symptoms and suggestive imaging findings. Median follow-up time was 46 months, ranging from 10 to 188 months.

Our results reinforce that a combined approach with anti-VEGF agents and anti-inflammatory therapy is effective in I-CNV treatment. In fact, in our study, most patients had a favourable visual outcome. Among the twenty included eyes, an approximate mean gain of 15 letters after anti-VEGF treatment (+15.10) was observed. Also, according to our classification, most had an improved visual acuity, with 16 eyes having gained 5 or more letters. Other 3 eyes maintained a stable visual outcome, with visual acuity not varying more than 4 letters (either a gain or a loss) from the pretreatment to the posttreatment stages. Additionally, 65% of eyes had a visual acuity equal to or greater than 65 letters at the end of the study, suggesting that the combined treatment approach is effective in maintaining visual improvement in the long term. Mansour et al. noted that a median of 3 injections of bevacizumab was able to sustain significant visual improvement in 8 eyes with I-CNV refractory to standard therapy at a 5-year interval [23].

Only 1 eye had an outcome classified as worsened (patient 13), with a loss of 8 letters. However, this individual had already been previously treated and followed in another medical centre. CNV was diagnosed in 2013 in his left eye and was treated with 5 injections until 2015. Before starting follow-up in our medical centre in January 2017, he had 3 other injections of aflibercept in June, October, and December 2016. Therefore, the pretreatment visual acuity measured in our medical centre does not represent the true pretreatment visual acuity of this patient. As a result, the difference in letters obtained in our study could potentially underestimate his real visual outcome and wrongly interpret the benefit of the instituted treatment measures.

Visual acuities did not differ between the infectious and the noninfectious subgroups. However, it is important to pinpoint the small number of cases belonging to the infectious subgroup (2 eyes), when compared with the noninfectious subgroup (18 eyes). This could explain the absence of statistical significance.

Complications seen in our sample were cataract and ocular hypertension. Cataract development correlated with a higher mean number of anti-VEGF intravitreal injections (35 *versus* 7, *p*=0.031).

In sum, our results show a favourable visual outcome for patients with I-CNV. However, we must emphasise the importance of regular screening. Intensive follow-up of both the inflammatory disease and the choroidal neovascular membrane is key to successively manage these conditions with the appropriate treatment, in order to promote a better visual prognosis and improve quality of life.

## 5. Strengths and Limitations

Since it is a retrospective study, we were able to observe the natural history and development of inflammatory choroidal neovascularization. This is essential since our aim was to describe a sample of patients, regarding their clinical characteristics, the treatments they underwent, and the evolution of both the disease itself and the corresponding visual acuity. Besides, due to the considerably extensive follow-up time, such characterisation was possible, and we could witness long-term phenomena such as recurrence and complications as well as the correspondent appropriate therapeutic manoeuvres.

Since we underwent an intensive search among virtually all inflammatory cases in our care centre (3076 cases in the initial list), we were able to positively ascertain that probably all I-CNV cases were included. Nevertheless, since it is an uncommon pathology and difficult to identify, we found only 17 patients, which is similar to peer case series studies regarding this theme.

Limitations were related to access and quality of gathered clinical data, pertaining to the retrospective nature of our study. However, it was possible to get the majority of the expected clinical information.

## 6. Conclusion

A combined treatment approach with anti-VEGF agents and systemic anti-inflammatory treatment was effective in the management of inflammatory choroidal neovascularization among eyes with various etiologies: punctate inner choroidopathy/multifocal choroiditis, sarcoidosis, serpiginous choroiditis, Vogt–Koyanagi–Harada disease, toxoplasmosis, and nocardiosis. Patients had an overall good visual prognosis, with improved or stable visual acuities after anti-VEGF treatment and prompt systemic immunomodulation. Rigorous follow-up was fundamental in the management of both the primary inflammatory and secondary neovascular conditions.

## Figures and Tables

**Table 1:** Infectious and noninfectious inflammatory choroidal neovascularization aetiologies.

Inflammatory choroidal neovascularization aetiologies	
*Noninfectious*	Multifocal choroiditis
	Serpiginous choroiditis
	Punctate inner choroidopathy
	Acute multifocal placoid pigment epitheliopathy
	Birdshot chorioretinitis
	Multiple evanescent white dot syndrome
	Vogt–Koyanagi–Harada syndrome
	Sympathetic ophthalmia
	Behcet's disease
	Sarcoidosis
	Multifocal choroiditis with panuveitis
	Idiopathic panuveitis
	Tubulointerstitial nephritis and uveitis

Infectious	Tuberculosis
	Toxoplasmosis
	West Nile virus
	Rubella retinopathy
	*Candida albicans*
	*Histoplasma capsulatum*
	*Cryptococcus neoformans*
	*Aspergillus fumigatus*
	Toxocara
	Endophthalmitis

*Note*. Adapted from [1].

**Table 2:** Sample description.

P	Age^a^	Sex	Inflammatory aetiology	I or NI	Inflammatory diagnosis date	CNV eyes	CNV location	CNV diagnosis date	Follow-up time^b^ (months)
P1	62	F	MFC/SERP	NI	March 16, 2005	L	SF	March 16, 2005	188
P2	27	F	PIC/MFC	NI	April 5, 2005	R	SF	April 5, 2005	186
P3	36	F	PIC/MFC	NI	May 7, 2012	R	SF	December 18, 2007	141
P4	32	M	PIC/MFC	NI	November 18, 2011	R	SF	November 5, 2009	126
P5	57	F	PIC/MFC	NI	March 9, 2012	R L	SF JF	July 13, 2010 January 4, 2017	123 46
P6	69	F	SARC	NI	1984	R	SF	August 23, 2012	76
P7	20	M	SERP	NI	November 1, 2013	R	JF	November 13, 2013	70
P8	28	F	PIC/MFC	NI	March 18, 2011	L	SF	June 18, 2014	71
P9	69	M	PIC/MFC	NI	2013^c^ (another centre)	L	PP	2013^c^ CHUSJ^d^: July 7, 2015	61
P10	53	F	PIC/MFC	NI	August 2015	R	SF	March 31, 2016	13^e^
P11	32	F	PIC/MFC	NI	June 12, 2015	L R	JF JF	August 16, 2016 September 15, 2016	44 43
P12	22	F	PIC/MFC	NI	October 1, 2016	L R	SF SF	November 9, 2016 May 24, 2019	40 9
P13	37	M	PIC/MFC	NI	2013^c^ (another centre)	L	SF	2013^c^ CHUSJ^d^: January 18, 2017	45
P14	57	F	SARC	NI	September 7, 2010	L	JF	March 22, 2017	42
P15	41	M	TOXO	I	November 2010	R	JF	June 6, 2017	40
P16	26	F	VKHD	NI	January 2016 (Brazil)	R	JF	February 8, 2018	23
P17	50	M	NOC	I	February 2016 (Germany)	L	SF	December 13, 2019	10

P: patient number; I: infectious aetiology; NI: noninfectious aetiology; F: female sex; M: male sex; PIC/MFC: punctate inner choroidopathy/multifocal choroiditis; SERP: serpiginous choroiditis; SARC: sarcoidosis; TOXO: toxoplasmosis; VKHD: Vogt–Koyanagi–Harada disease; NOC: nocardiosis; SF: subfoveal location; JF: juxtafoveal location; PP: peripapillary location. ^a^Age of CNV diagnosis; ^b^from CNV diagnosis until last examination; ^c^previous diagnosis and follow-up in another medical centre; ^d^beginning of follow-up in CHUSJ in cases previously diagnosed; ^e^lost to follow-up: last consultation on May 2, 2017.

**Table 3:** Treatment.

Eye	CNV eye	Total anti-VEGF injections	Anti-VEGF agents^a^ (number of injections)	Anti-VEGF status^b^	Systemic treatment	Treatment for TB	Topic treatments
E1	L	111	R (24) B (68) A (19)	M	PDT CCTs (p.o.)	HRZE	CCTs
E2	R	2	B (2)	E	PDT CCTs (p.o.)	No	No
E3	R	6	B (6)	E	—	No	CCTs NSAIDs
E4	R	38	R (3) B (35)	M	CCTs (p.o.) Cyclosporin Azathioprine Adalimumab	Isoniazid (prophylaxis before biologic therapy)	CCTs NSAIDs For OHTN
E5.1	R	35	R (5) B (18) A (12)	M	—	HRZE	CCTs NSAIDs
E5.2	L	42	B (30) A (12)	M	—	HRZE	CCTs NSAIDs
E6	R	10	B (10)	E	CCTs (p.o.)	No	CCTs NSAIDs For OHTN
E7	R	3	B (3)	E	—	No	No
E8	L	2	B (2)	E	CCTs (p.o. and intravitreal: triamcinolone) Cyclosporin	No	CCTs NSAIDs
E9	L	5	A (2) B (3)	E	—	No	No
E10	R	11	B (3) A (3) R (5)	E	—	No	No
E11.1	L	7	B (7)	E	CCTs (p.o.) Cyclosporin	No	For OHTN
E11.2	R	7	B (7)	E	CCTs (p.o.) Cyclosporin	No	For OHTN
E12.1	L	4	B (4)	E	CCTs (p.o.) Cyclosporin	No	CCTs NSAIDs
E12.2	R	3	B (3)	E	CCTs (p.o.) Cyclosporin	No	No
E13	L	37	B (5) A (3) B (29)	M	CCTs (p.o.) Cyclosporin	No	No
E14	L	6	B (6)	E	CCTs (p.o.) Methotrexate	No	CCTs For OHTN
E15	R	7	B (7)	M	—	No	No
E16	R	10	B (10)	E	CCTs (p.o. and intravenous)	No	No
E17	L	7	B (4) A (3)	M	TMP-SMX + *β*-lactam	No	No

^a^Ordered from oldest to most recent agent used in each eye; ^b^anti-VEGF status at final data collection (November 2020): M: maintained anti-VEGF treatment or E: ended anti-VEGF treatment; R: right; L: left; B: bevacizumab; A: aflibercept; R: ranibizumab; PDT: photodynamic therapy; OHTN: ocular hypertension; CCTs: corticosteroids; NSAIDs: nonsteroidal anti-inflammatory drugs; p.o.: *per os*; TMP-SMX: trimethoprim-sulfamethoxazole; TB: tuberculosis; HRZE: isoniazid (H) + rifampicin (R) + pyrazinamide (Z) + ethambutol (E).

**Table 4:** Visual outcomes.

	Pre-Tx VA	Post-Tx VA	ΔVA	*p*
Mean	50.85	65.95	+15.10	0.000051
SD	19.148	19.168	12.998	

**Table 5:** Visual acuities and visual outcomes.

Eye	Age^a^	Sex	Inflammatory aetiology	CNV eye	Baseline VA	Pre-Tx VA	Post-Tx VA	Final VA	ΔVA	Visual outcome^d^
E1	62	F	MFC/SERP	L	65	38	38^c^	38^c^	0	Stable
E2	27	F	PIC/MFC	R	55	58	59	58	1	Stable
E3	36	F	PIC/MFC	R	30	53	82	82	29	Improved
E4	32	M	PIC/MFC	R	65^b^	65^b^	76	30	11	Improved
E5.1	57	F	PIC/MFC	R	19^b^	19^b^	68^c^	68^c^	49	Improved
E5.2	63	F	PIC/MFC	L	74	37	68^c^	68^c^	31	Improved
E6	69	F	SARC	R	35	33	50	45	17	Improved
E7	20	M	SERP	R	85	72	84	83	12	Improved
E8	28	F	PIC/MFC	L	80	52	74	81	22	Improved
E9	69	M	PIC/MFC	L	50^b^	50^b^	62	70	12	Improved
E10	53	F	PIC/MFC	R	35	31	30	30	−1	Stable
E11.1	32	F	PIC/MFC	L	70	50	76	75	26	Improved
E11.2	32	F	PIC/MFC	R	26	15	20	15	5	Improved
E12.1	22	F	PIC/MFC	L	85	67	85	84	18	Improved
E12.2	25	F	PIC/MFC	R	85	70	80^c^	80^c^	10	Improved
E13	37	M	PIC/MFC	L	84^b^	84^b^	76^c^	76^c^	−8	Worsened
E14	57	F	SARC	L	80	54	73	67	19	Improved
E15	41	M	TOXO	R	85	73	85^c^	85^c^	12	Improved
E16	26	F	VKHD	R	70	66	83	84	17	Improved
E17	50	M	NOC	L	65	30	50^c^	50^c^	20	Improved

F: female sex; M: male sex; R: right; L: left; PIC/MFC: punctate inner choroidopathy/multifocal choroiditis; SERP: serpiginous choroiditis; SARC: sarcoidosis; TOXO: toxoplasmosis; VKHD: Vogt–Koyanagi–Harada disease; NOC: nocardiosis. ^a^Age of CNV diagnosis; ^b^baseline VA was equal to pretreatment VA in patients when primary inflammatory and I-CNV presentations were simultaneous; ^c^final VA was equal to posttreatment VA in patients that maintained intravitreal anti-VEGF treatment at the last evaluation; ^d^an outcome higher than or equal to 5 letters was considered improved, an outcome between −4 and 4 letters was counted as stable, and an outcome equal to or lower than −5 letters was classified as worsened.

## Data Availability

All the data used to support the findings of this study are included within the article.

## Supplementary Materials

Supplementary Materials Supplementary Table 1: medical history. Supplementary Table 2: recurrence and treatment suspension. Supplementary Table 3: complications. .
