# Supplementary material for: Inflammatory Choroidal Neovascular Membranes: Clinical Profile, Treatment Effectiveness, and Visual Prognosis
Source: J Ophthalmol. 2021 Jul 23;2021:9982883. doi: 10.1155/2021/9982883 (PMC8324381; doi:10.1155/2021/9982883)
Supplement: Supplementary Materials — Supplementary Table 1: medical history. Supplementary Table 2: recurrence and treatment suspension. Supplementary Table 3: complications. . [file 9982883.f1.docx]

## Supplementary Material

**SUPPLEMENTARY TABLE** **1** – MEDICAL HISTORY

**SUPPLEMENTARY TABLE 2** – RECURRENCE AND TREATMENT SUSPENSION

**SUPPLEMENTARY TABLE 3–** COMPLICATIONS

**SUPPLEMENTARY TABLE** **1** – MEDICAL HISTORY

| Patient  Age^a^  Sex  Inflammatory aetiology | Other ophthalmologic history | Eye refraction | General history | Surgeries | Regular Medication |
| --- | --- | --- | --- | --- | --- |
| P1  77  F  MFC/SERP | - Cataract R and L eyes - Photodynamic therapy (2005) | R: +1.00  L: +2.00 | - Arterial hypertension - Dyslipidaemia - Atrial Fibrillation - Asthma | - Bladder surgery - Basal cell carcinoma in inferior R eyelid (surgical excision January 2013) | - Acetylsalicylic acid - Warfarin - Bisoprolol - Pravastatin |
| P2  43  F  PIC/MFC | - High myopia - Photodynamic therapy (2005) | R: – 12.50  L: – 11.00 | - Fibromyalgia - Gastroduodenal perforation | - Gastroduodenal perforation surgery (2016) | - Pregabalin - Duloxetine - Fentanyl |
| P3  49  F  PIC/MFC | - High myopia - Cataract R eye (incipient) - Cataract L eye (incipient) | R: –13.75  L: –13.00 | - None | - None | - None |
| P4  44  M  PIC/MFC | - Monocular with R eye (L eye perforation at 6 years old, with secondary ocular hypertension) - High myopia - Cyclophotocoagulation (2005) | R: –19.75  L: NA  (perforating eye trauma) | - Dyslipidaemia - Depression - Acute gastroenteritis - Luxated knee - Luxated finger (2nd, left hand) - Latent tuberculosis (primo infection in infancy and treated) | - Cholecystectomy - Appendectomy - Tonsillectomy - Umbilical hernia repair - L eye evisceration and prothesis replacement (May 25, 2012) | - Pantoprazole - Atorvastatin - Sertraline |
| P5  67  F  PIC/MFC | - Ocular tuberculosis R eye (2012) - High myopia - Cataract R and L eyes (operated) | R: –14.00  L: –14.00 | - Arterial hypertension - Dyslipidaemia - Insomnia - Uterine polyps - Head injury | - Elbow fracture (operated) - Uterine polyps hysteroscopic resection - Phacoemulsification with intraocular lens implantation | - Ramipril - Simvastatin - Zolpidem |
| P6  78  F  SARC | - Cataracts R and L eyes (operated) - Ocular hypertension (glaucoma, cyclophotocoagulation L eye and iridectomy L and R eyes) - Monocular R eye (loss of vision L eye) | NA  (operated previously in another medical centre, no info) | - Arterial hypertension - Dyslipidaemia - Type 2 diabetes - Hypothyroidism (multinodular goiter) - Hepatic sarcoidosis - Pulmonary sarcoidosis - Irritable bowel syndrome | - Breast lump removal - Cholecystectomy - Hysterectomy - Appendectomy - Pancreatic - Knee prothesis (L) - Phacoemulsification with intraocular lens implantation | - Valsartan + hydrochlorothiazide - Glicazide - Simvastatin - Acetylsalicylic acid - Budesonide - Formoterol - Long-term oxygen therapy - Levothyroxine |
| P7  27  M  SERP | - None other | R: 0.00  L: 0.00 | - Acute tonsillitis - Infectious gastroenteritis | - None | - None |
| P8  35  F  PIC/MFC | - High myopia | R: –9.00  L: –11.00 | - None | - None | - None |
| P9  76  M  PIC/MFC | - Bilateral LASIK surgery (2005) | NA  (previous LASIK surgery) | - Arterial hypertension - Type 2 diabetes - Gastroesophageal reflux disease - Presbycusis (implants) | - Bilateral LASIK surgery (2005) - Ankle fracture (operated) | - Metformin - Valsartan + hydrochlorothiazide - Omeprazole |
| P10  58  F  PIC/MFC | - No info | R: 0.00  L: –0.50 | - None | - None | - None |
| P11  37  F  PIC/MFC | - High myopia - Ocular hypertension | R: –14.00  L: –14.00 | - None | - None | - None |
| P12  26  F  PIC/MFC | - High myopia | R: –9.00  L: –10.00 | - Asthma/Bronchitis - Depression - Insect sting allergy reaction - Hansen's disease/Leprosy (2009) (tuberculoid/ paucibacillary; *Mycobacterium leprae*) | - None | - Sodium valproate - Venlafaxine - Tiapride |
| P13  45  M  PIC/MFC | - Bilateral LASIK surgery (2007) - Cataract L eye (incipient) | R: –5.00  L: –5.00 | - None (previously followed in another medical centre) | - Bilateral LASIK surgery (2007) | - None |
| P14  61  F  SARC | - Ocular hypertension (chronic glaucoma) - Cataract L and R (operated) | R: –5.00  L: –1.75 | - Dyslipidaemia - Hepatic steatosis - Sarcoidosis (multiorgan involvement: bilateral posterior uveitis, cutaneous involvement, bilateral hilar and mediastinal lymphadenopathy) - Asthmatic bronchitis - Lumbar herniated disk - Breast cancer (right; 2004) - Hypothyroidism (autoimmune thyroiditis) | - Phacoemulsification with intraocular lens implantation | - Levothyroxine - Atorvastatin - Omeprazole - Folic acid |
| P15  44  M  TOXO | - None other | R: –1.75  L: –1.75 | - Sprained ankle - Acute sphenoid sinusitis - Nasal septum deviation (posterior) - Hepatic steatosis | - Nasal septum resection | - None |
| P16  29  F  VKHD | - None other | R: 0  L: –0.25 | - Lichen planus - Osteopenia - Vitiligo | - None | - Calcium - Vitamin D |
| P17  51  M  NOC | - None other | R: –0.25  L: 0 | - Systemic nocardiosis (pulmonary, cerebral, cutaneous): Nocardia abscessus - Benign prostatic hyperplasia - Epilepsy (post cerebral abscess) - Pulmonary silicosis | - None | - Furosemide - Tamsulosin - Levetiracetam - Trazodone - Glycopyrronium bromide + indacaterol maleate |

P – Patient number; ^a^Age at final data collection (November 2010); PIC/MFC – Punctate Inner Choroidopathy/Multifocal Choroiditis; SERP – Serpiginous Choroiditis; SARC – Sarcoidosis; TOXO – Toxoplasmosis; VKHD – Vogt-Koyanagi-Harada Disease; NOC – Nocardiosis; F – Female sex; M – Male sex; R – Right; L – Left; NA – Not applicable; LASIK - Laser-assisted in situ keratomileusis

**SUPPLEMENTARY TABLE** **2** – RECURRENCE AND TREATMENT SUSPENSION

| Eye | Age^a^; Sex Inflammatory aetiology | Inflammatory Recurrence | Treatment for Inflammatory recurrence | CNV recurrence | Treatment for CNV recurrence | Anti-VEGF suspension |
| --- | --- | --- | --- | --- | --- | --- |
| E1 | 62; F  MFC/SERP | Yes | CCTs (p.o.) | No | - | Never suspended (R > B > A) |
| E2 | 27; F  PIC/MFC | No | - | Yes | B | Periods of suspension |
| E3 | 36; F  PIC/MFC | No | - | No | - | Periods of suspension |
| E4 | 32; M  PIC/MFC | Yes | CCTs (p.o.)  Cyclosporin  Azathioprine Adalimumab | No | - | Never suspended (R > B) |
| E5.1 | 57; F  PIC/MFC | No | - | Yes | B | Periods of suspension |
| E5.2 | 63; F  PIC/MFC | No | - | No | - | Never suspended (B) |
| E6 | 69; F  SARC | No | - | No | - | Periods of suspension |
| E7 | 20; M  SERP | No | - | No | - | Periods of suspension |
| E8 | 28; F  PIC/MFC | Yes | Systemic CCT (p.o.)  Cyclosporin  Triamcinolone | No | - | Periods of suspension |
| E9 | 69; M  PIC/MFC | No | - | No | - | Periods of suspension |
| E10 | 53; F  PIC/MFC | No | - | No | - | Periods of suspension (lost follow up) |
| E11.1 | 32; F  PIC/MFC | No | - | No | - | Periods of suspension |
| E11.2 | 32; F  PIC/MFC | No | - | No | - | Periods of suspension |
| E12.1 | 22; F  PIC/MFC | No | - | No | - | Periods of suspension |
| E12.2 | 25; F  PIC/MFC | No | - | No | - | Periods of suspension |
| E13 | 37; M  PIC/MFC | Yes | Systemic CCT (p.o.) | No | - | Never suspended (B) |
| E14 | 57; F  SARC | No | - | No | - | Periods of suspension |
| E15 | 41; M  TOXO | No | - | Yes | B | Periods of suspension |
| E16 | 26; F  VKHD | No | - | No | - | Periods of suspension |
| E17 | 50; M  NOC | No | - | No | - | Never suspended (recent diagnosis: 2019, B > A) |

PIC/MFC – Punctate Inner Choroidopathy/Multifocal Choroiditis; SERP – Serpiginous Choroiditis; SARC – Sarcoidosis; TOXO – Toxoplasmosis; VKHD – Vogt-Koyanagi-Harada Disease; NOC – Nocardiosis; B – Bevacizumab; A – Aflibercept; R – Ranibizumab; CCTs – Corticosteroids; p.o. – *Per os*; ^a^Age of CNV diagnosis

**SUPPLEMENTARY TABLE** **3** – COMPLICATIONS

| Eye | Age^a^; Sex; Inflammatory aetiology | Cataract | Cataract surgery^b^ and date | Ocular Hypertension |
| --- | --- | --- | --- | --- |
| E1 | 62; F  MFC/SERP | Nuclear cataract R  Nuclear cataract L | No | No |
| E2 | 27; F  PIC/MFC | No | No | No |
| E3 | 36; F  PIC/MFC | Cataract R Cataract L | No | No |
| E4 | 32; M  PIC/MFC | No | No | Yes (topic medication) |
| E5.1 | 57; F  PIC/MFC | Cataract R Cataract L | R: July 12, 2019  L: October 18, 2019 | No |
| E5.2 | 63; F  PIC/MFC | Cataract R Cataract L | R: July 12, 2019  L: October 18, 2019 | No |
| E6 | 69; F  SARC | Cataract R Cataract L | R and L: before 2011 (exact date not available) | Yes (topic medication, trabeculectomy, cyclophotocoagulation) |
| E7 | 20; M  SERP | No | No | No |
| E8 | 28; F  PIC/MFC | No | No | No |
| E9 | 69; M  PIC/MFC | No | No | No |
| E10 | 53; F  PIC/MFC | No | No | No |
| E11.1 | 32; F  PIC/MFC | No | No | Yes (topic medication) |
| E11.2 | 32; F  PIC/MFC | No | No | Yes (topic medication) |
| E12.1 | 22; F  PIC/MFC | No | No | No |
| E12.2 | 25; F  PIC/MFC | No | No | No |
| E13 | 37; M  PIC/MFC | Posterior subcapsular cataract L (incipient) | No | No |
| E14 | 57; F  SARC | Cataract R  Cataract L | R: October 30, 2015  L: October 23, 2015 | Yes (topic medication) |
| E15 | 41; M  TOXO | No | No | No |
| E16 | 26; F  VKHD | No | No | No |
| E17 | 50; M  NOC | No | No | No |

F – Female sex; M – Male sex; R – Right; L – Left; PIC/MFC – Punctate Inner Choroidopathy/Multifocal Choroiditis; SERP – Serpiginous Choroiditis; SARC – Sarcoidosis; TOXO – Toxoplasmosis; VKHD – Vogt-Koyanagi-Harada Disease; NOC – Nocardiosis; ^a^Age of CNV diagnosis; ^b^Phacoemulsification with intraocular lens implantation
